# The Sorption Performance of Cetyl Trimethyl Ammonium Bromide-Capped La_0.9_Sr_0.1_FeO_3_ Perovskite for Organic Pollutants from Industrial Processes

**DOI:** 10.3390/molecules25071640

**Published:** 2020-04-02

**Authors:** Shimaa M. Ali, Areej A. Eskandrani

**Affiliations:** 1 Chemistry Department, Faculty of Science, Cairo University, Giza 12613, Egypt; 2 Chemistry Department, Faculty of Science, Taibah University, Medinah 30002, Saudi Arabia; a.eskandrani@gmail.com

**Keywords:** Ternary perovskites, surfactant, capping, adsorption, Congo red dye, water decontamination

## Abstract

La_0.9_Sr_0.1_FeO_3_ perovskite, prepared by the microwave-assisted method, was capped with cetyl trimethyl ammonium bromide (CTAB) cationic surfactant, and applied as a sorbent for the removal of the anionic Congo red (CR) dye from aqueous solutions. X-ray diffraction (XRD) patterns showed that the perovskite structure was not affected by capping; however, the particle size increased. There was a hipsochromic shift in the value of λ_max_ of the CR absorption spectrum in the presence of CTAB, which indicated the formation of an oppositely charged dye–surfactant complex. The adsorption efficiency of CTAB-capped La_0.9_Sr_0.1_FeO_3_ was independent of the pH of the solution—equilibrium was reached after a few minutes. The value of the maximum adsorption capacity, *q_m_*, was 151.52 mg·g^−1^, which was 10-times higher than that of the pure perovskite. The proposed sorbent maintained its excellent sorption ability in the presence of the sample matrix; therefore, it can be regenerated and reused with unchanged performance.

## 1. Introduction

Surfactants are organic compounds that can be used to decrease the surface tension of liquids. A surfactant consists of both hydrophilic (heads) and hydrophobic (tails) groups; therefore, it can be used in detergents, wetting and foaming agents, dispersants, etc. It can be classified according to the charge of its hydrophilic groups into cationic, anionic, non-ionic, and Zwitter-ionic surfactants [1]. Surfactants have various important applications in many fields, they can be added during the synthesis of nanoparticles to prevent coagulation, since they act as a stabilizer. This is particularly important in the synthesis of magnetic nanoparticles [2,3,4,5]. Surfactants and their composites can be used for water and wastewater decontamination, where they can be used to remove oppositely charged toxic metal ions and organic compounds via strong electrostatic attractions [6,7,8]. They can also be used for the corrosion protection of metals that can from an oxide layer, where surfactants can be adsorbed onto the oxide layer, providing a protective coating for the metal [9,10,11]. Surfactants were also used to improve the electrochemical sensing performance of many compounds and drugs [12,13,14,15] and catalytic processes [16,17,18,19,20].

Perovskites are mixed nano-oxides of the general formula ABO_3_, where A is a lanthanide or an alkali earth metal and B is a transition metal. Perovskites are interesting materials because a wide range of properties can result from a large number of possible metal ion combinations that can form the perovskite structure [21,22]. Perovskites have several important applications, for example in catalysis, sensors [23,24,25], and as sorbents for water decontamination [26,27,28].

Organic dyes constitute a major source of pollution in wastewater produced from many industries, which poses a threat to the water quality and the life of living beings [29]. The removal of organic dyes using perovskite sorbents was reported in the literature. SrTiO_3_ nanoparticles were used for the removal of the anionic congo red (CR) [30], and the cationic malachite green dye [31]. Silica-coated LaMnO_3_ perovskite was used for the removal of cationic methylene blue and anionic methyl orange dyes [32]. La_0.5_Pb_0.5_MnO_3_ [33] and La_0.5_Ca_0.5_NiO_3_ [34] were used for the removal of Eosin dye and reactive blue, respectively.

Cetyl trimethyl ammonium bromide (CTAB) is a cationic surfactant, CTAB-modified materials can be used for the removal of the anionic CR dye from aqueous solutions, such as CTAB/chitosan [35], CTAB/chitosan hydrogel beads [36], CTAB/tea waste [37], CTAB/hectorite [38], CTAB/kaolin [39], CTAB/bentonite [40], and CTAB/graphene oxide [41]. 

Most perovskites have a negative charge under neutral conditions [26]; therefore, a cationic surfactant can be easily adsorbed onto the perovskite surface and used for the removal of negatively charge ions or anionic dyes from water. The combination of CTAB and perovskites has not been previously reported. Therefore, in this work, La_0.9_Sr_0.1_FeO_3_ perovskite, prepared by the microwave-assisted method, will be capped by CTAB and then employed as an adsorbent for CR dye from aqueous solutions and real samples. Factors affecting the adsorption process such as pH, contact time, initial dye concentration, and temperature will be studied and optimized. The sorption performance of CTAB-capped La_0.9_Sr_0.1_FeO_3_ perovskite is compared with the pure perovskite and with other reported CTAB-capped materials. A method for the sorbent regeneration and reuse is examined. 

## 2. Results and Discussions

### 2.1. Characterization of CTAB-capped La_0.9_Sr_0.1_FeO_3_ Perovskite

**Figure 1:**
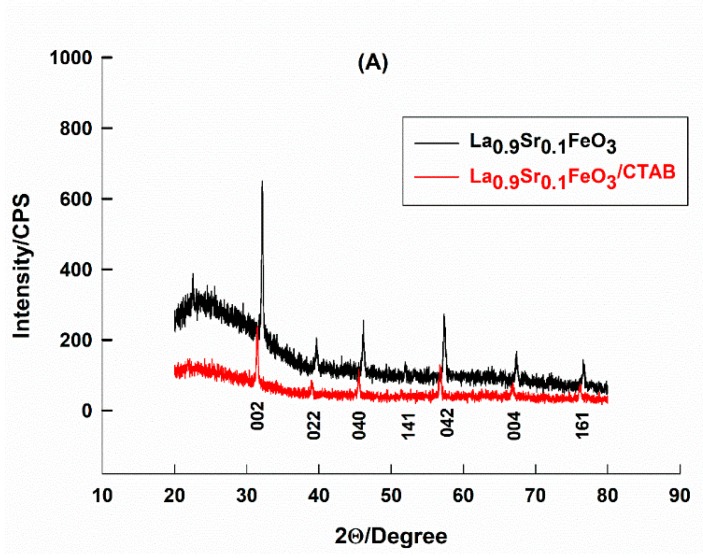

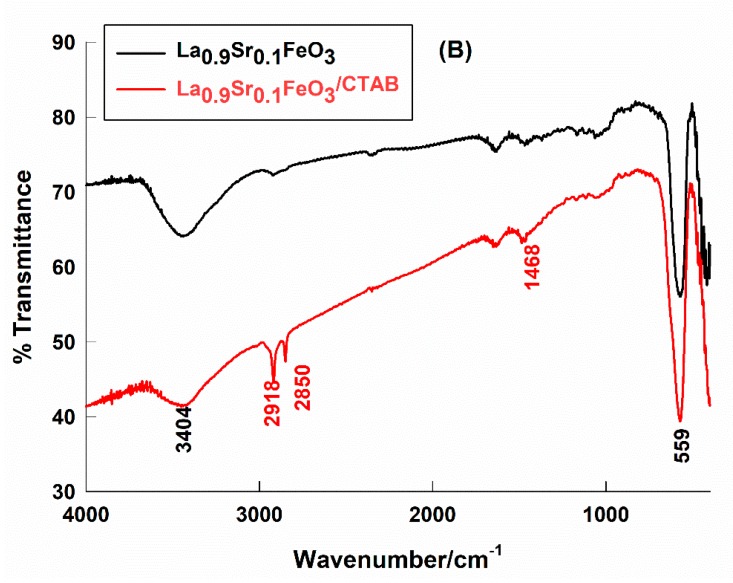
XRD patterns (**A**) and FTIR spectra (**B**) of pure and CTAB-capped La_0.9_Sr_0.1_FeO_3_. Corresponding Miller indices and wavenumbers are indicated.

Structural characterizations of pure and CTAB-capped La_0.9_Sr_0.1_FeO_3_ perovskites were carried out by XRD and FTIR, to show the effect of surfactant capping on the perovskite structure and to prove that the modification was successfully performed. Figure 1A shows XRD patterns of pure and CTAB-capped samples. Similar XRD patterns were noticed for both samples, indicating that surfactant capping did not affect the perovskite structure. By comparing data to the standard LaFeO_3_, ICDD card number: 88-641, all diffraction peaks were indexed to corresponding planes, as indicated in Figure 1A. 

The phase identification showed that a single orthorhombic phase of LaFeO_3_ was formed, with higher d-values than those of the standard LaFeO_3_ sample. This was due to the partial replacement of the smaller La^3+^ ions with the larger Sr^2+^ ions [26]. However, two differences can be observed between XRD spectra of pure and CTAB-capped perovskites, peaks were narrower and shifted to higher d-values due to the surfactant modification, which indicated that the particle size is higher in the CTAB-capped sample compared to that in pure CTAB. The calculated average particle sizes, using the Schererr equation [42] were 30.8, 45.3 nm, for pure and CTAB-capped samples, respectively. The second difference is the lower peak intensities in the case of the CTAB-capped sample, which indicated that there was an interaction between the perovskite and CTAB, this interaction was also indicated in the FTIR data.

Figure 1B shows FTIR spectra of pure and CTAB-capped La_0.9_Sr_0.1_FeO_3_ perovskites. The characteristic Fe-O stretching band of the perovskite FeO_6_ octahedral group appeared in both samples at 559 cm^−1^ [26]. Furthermore, a strong band at 3404 cm^−1^ can be observed, which was assigned to the O-H stretching vibration of adsorbed water. Additional bands at 2918 and 2850 cm^−1^ appeared only in the FTIR spectrum of the CTAB-capped sample. These bands were due to the C-H stretching vibration of -CH_3_ and -CH_2_ groups of CTAB, respectively. A weak band appeared at 1468 cm^−1^, which was assigned to N^+^-CH_3_ absorption—this band had a strong signal in the FTIR spectrum of pure CTAB [6,43]. However, in the case of the CTAB-capped perovskite FTIR spectrum, the intensity of this band was weak, which indicated that there was an interaction between the perovskite and CTAB through its ammonium moiety. The appearance of these bands proved that the CTAB surfactant modification of La_0.9_Sr_0.1_FeO_3_ perovskite was successful. At a pH value > the point of zero charge, the perovskite acquired negative charges, which can attract the ammonium moieties in CTAB, resulting in the CTAB-capped perovskite [26,31]. 

The measured BET surface area values for pure and CTAB-capped La_0.9_Sr_0.1_FeO_3_ were 3.9 and 2.3 m^2^·g^−1^, respectively. The CTAB modification decreased the perovskite surface area. 

**Figure 2:**
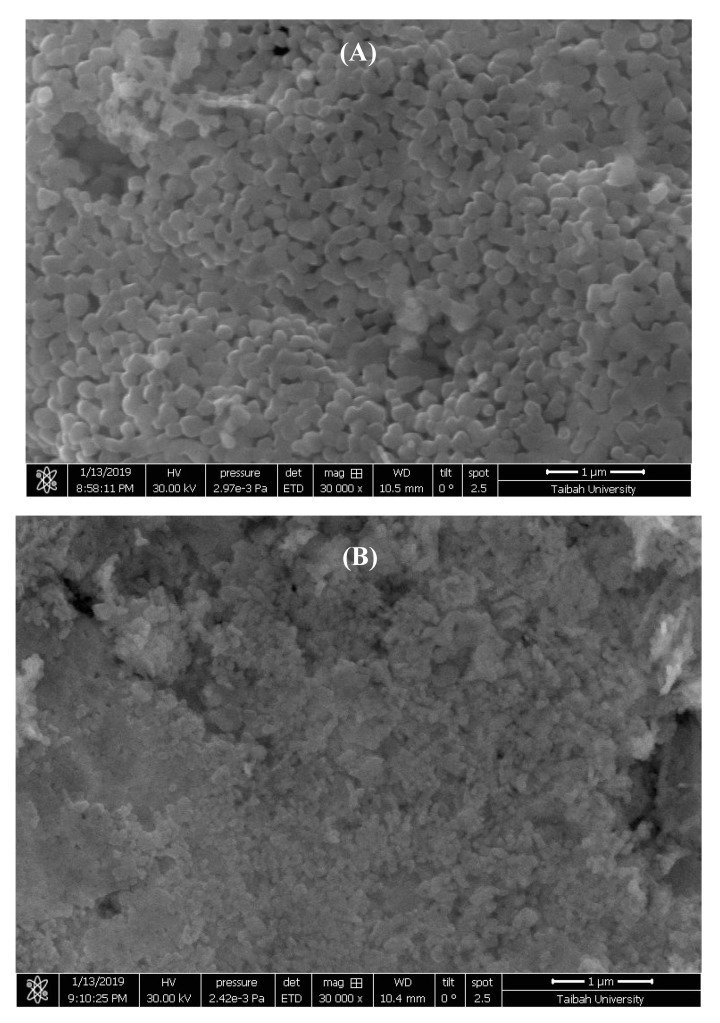
SEM images of pure (**A**), and CTAB-capped La_0.9_Sr_0.1_FeO_3_ (**B**), with 30,000 times magnification.

The surface morphology of prepared samples was studied by SEM. Figure 2 shows SEM images of pure and CTAB-capped La_0.9_Sr_0.1_FeO_3_ samples. The pure La_0.9_Sr_0.1_FeO_3_ consisted of an interconnected bone-like network, Figure 2A. Upon the surfactant modification, SEM imaging, Figure 2B, shows that perovskite grains were more conjoined, thereby reducing interfacial spaces, as compared to the pure sample, Figure 2A. This clearly showed the decreased porosity and, therefore, the decreased surface area of the perovskite by the modification with CTAB.

### 2.2. Application of CTAB-capped La_0.9_Sr_0.1_FeO_3_ Perovskite as a Sorbent for CR Dye

**Figure 3:**
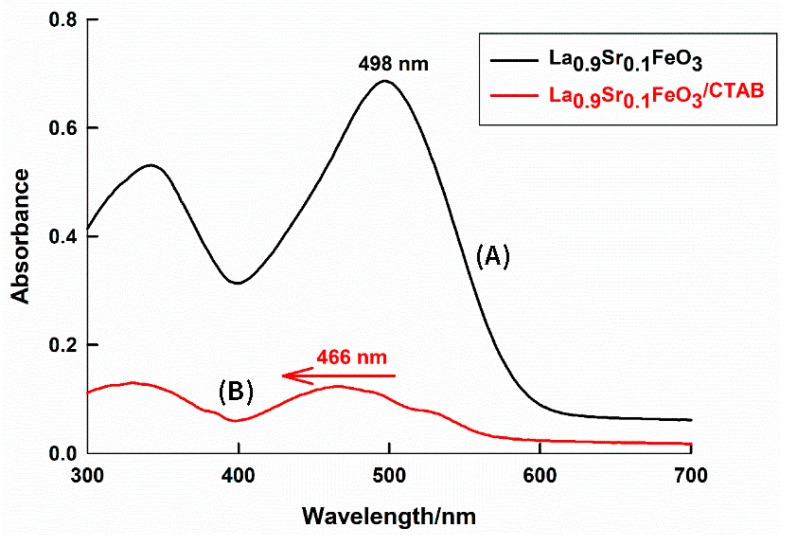
Visible absorption spectra of remaining CR dye in solutions by using (**A**) pure, and (**B**) CTAB-capped La_0.9_Sr_0.1_FeO_3_ sorbents at pH = 6, the dye concentration = 10 ppm, contact time = 24 h, at 25 °C.

The absorption characteristic of the non-adsorbed CR dye in the solution was different when different sorbents were used—pure and CTAB-capped La_0.9_Sr_0.1_FeO_3_ perovskites. Figure 3 shows the visible spectrum of the remaining CR dye, non-adsorbed in the solution, after batch experiments were performed using different sorbents. In the case of using the pure La_0.9_Sr_0.1_FeO_3_, the visible spectrum was normal with a maximum wavelength, λ_max_, of 498 nm, as reported in the literature [26]. On the other hand, the use of CTAB-capped La_0.9_Sr_0.1_FeO_3_ resulted in a decreased λ_max_ value, at 466 nm, showing a hipsochromic shift. CR dye is an anionic dye, while CTAB is a cationic surfactant; therefore, an oppositely charged dye–surfactant complex is formed. This can cause a dye dimerization in the presence of the surfactant and a decrease in the dye absorbance value. It was reported that the dye dimerization could occur at a high dye concentration or in the presence of large molecules [44]. It can be seen that in the presence of CTAD—the surfactant-capped perovskite—the absorbance value of the non-adsorbed CR dye was largely decreased compared to that of the pure perovskite sorbent, which reflected the enhanced sorption ability of La_0.9_Sr_0.1_FeO_3_ for the anionic CR dye as a result of being capped with a cationic CTAB surfactant.

#### 2.2.1. Effect of pH on the Adsorption Performance

**Figure 4:**
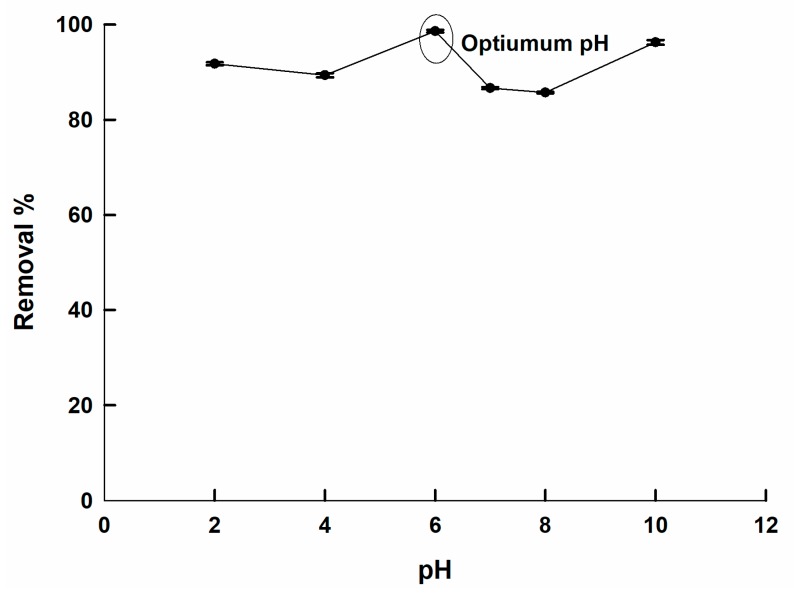
The relation between the CR removal % of by CTAB-capped La_0.9_Sr_0.1_FeO_3_ and the solution pH, the dye concentration = 30 ppm, contact time = 24 h, at 25 °C.

It is well known that the pH value can greatly affect the removal efficiency of a sorbent. For most perovskites, the point of zero charge is about 5 [26,31]. Therefore, the perovskite is positively charged at pH < 5, and it is expected to have its highest sorption ability for the anionic dye under acidic conditions. Figure 4. shows the dependence of the removal % of CTAB-capped La_0.9_Sr_0.1_FeO_3_ for CR on the pH of the dye solution. It can be shown that the adsorption performance was independent of the pH. A high value of the removal % was noticed at any pH value. This can be explained on the basis that the positively charged CTAB-perovskite sorbent can attract the negatively charged CR dye regardless of the pH value. Therefore, the proposed sorbent is suitable to be used in applications as it possesses a superior performance, irrespective of the operating pH. In the next sections, the optimum pH value will be taken to be 6, since it is close to neutral conditions and shows a removal % of 98.8%.

#### 2.2.2. Kinetic Study

**Figure 5:**
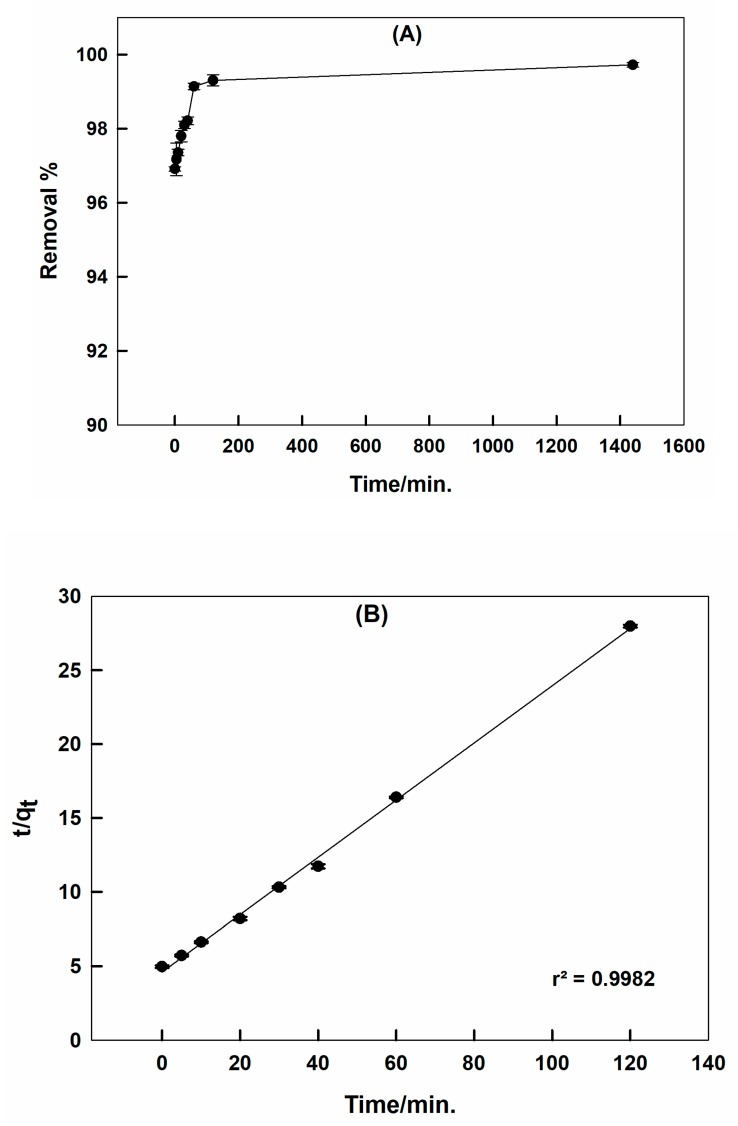
The relation between the CR removal % by CTAB-capped La_0.9_Sr_0.1_FeO_3_ and the contact time; pH = 6, the dye concentration = 30 ppm, at 25 °C (**A**), and pseudo 2nd order model (**B**).

The effect of the contact time was examined by estimating the adsorbed dye concentrations at different times, extended to 2 h, to identify the equilibrium position and to investigate the kinetics of the adsorption process. Figure 5A shows the relation between the removal % of CR by CTAB-capped La_0.9_Sr_0.1_FeO_3_ and the contact time. It can be shown that the equilibrium is reached very fast, the removal % is ~97% at the start of the experiment. This reflected the possibility of applying the present sorbent in the field use, as it offered a rapid dye uptake.

The 1st and 2nd order models are given by Equations (1) and (2), respectively [45,46,47,48]:

(1) $$\log\left(q_{e}-q_{t}\right)=logq_{e}-\frac{K_{1}}{2.303}t$$

(2) $$\frac{t}{q_{t}}=\frac{1}{K_{2}q_{e}}+\frac{t}{q_{e}}$$

where *q_e_*, *q_t_* were the adsorbed amounts in mg·g^−1^ at equilibrium, and at time t (min), respectively. *K*_1_ and *K*_2_ were first- and second-order rate constants, respectively.

It was found that the adsorption data did not fit the pseudo 1st order model at all, while it perfectly fitted the pseudo 2nd order model, as shown in Figure 5B. This implied that the surface reaction between the positively charged surfactant-capped perovskite and the negatively charged dye was the rate-determining step rather than the adsorption of dye on the sorbent active sites. In addition, it reflected the possibility of a dynamic equilibrium between the adsorbate and surface sites during the diffusion through the sorbent pores [49]. The experimental *q_e_* value calculated from the pseudo 2nd order model matches well with the theoretical value, 5.21 and 5.16 mg·g^−1^, respectively. The calculated value of the rate constant, *K*_2_, was 0.42 g·mg^−1^·min^−1^.

#### 2.2.3. The Effect of the Initial Dye Concentration

**Figure 6:**
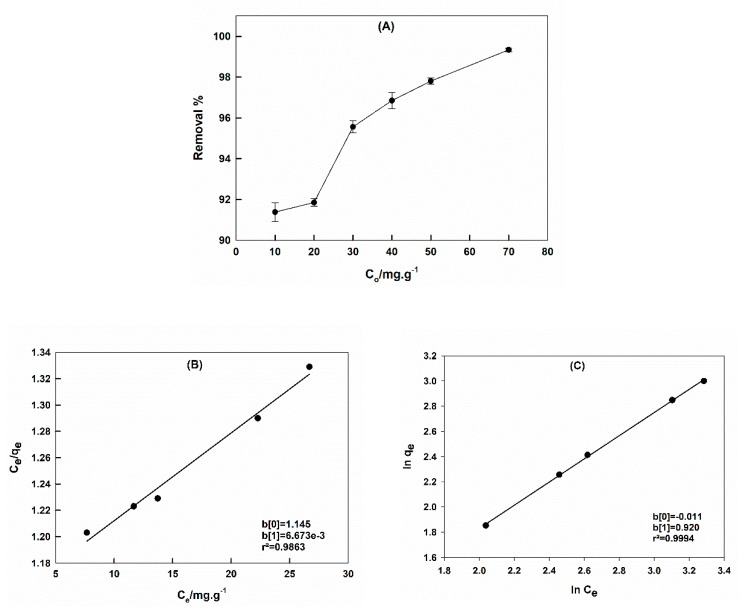
The relation between the CR removal by CTAB-capped La_0.9_Sr_0.1_FeO_3_ and the initial CR concentration; pH = 6, contact time = 1 h, at 25 °C (**A**). Langmuir (**B**) and (**C**) Freundlich isotherms.

Batch experiments were conducted by using different initial dye concentrations, 10–100 ppm, to examine the effect of the dye concentration. Figure 6A shows the relation between the removal % and the initial concentration of CR dye; the removal % increased as the dye concentration increased.

Adsorption data were fitted to Langmuir and Freundlich isotherms to deduce the mechanism of adsorption and to estimate the maximum adsorption capacity, *q_m_*, of CTAB-capped La_0.9_Sr_0.1_FeO_3_ for CR dye. Langmuir and Freundlich isotherms can be expressed by the following equations, respectively [35,45,46,47,48]:

(3) $$\frac{C_{e}}{q_{e}}=\frac{1}{q_{m}b}+\frac{C_{e}}{q_{m}}$$

(4) $$lnq_{e}=lnK_{f}+\frac{1}{n}lnC_{e}$$

where *b* was Langmuir constant, *K_f_* and n were Freundlich isotherm constants.

Figure 6B,C represent Langmuir and Freudlich isotherms, respectively. Correlation coefficient values were 0.9863 and 0.9994, respectively. This showed that the adsorption data were better fitted using the Freundlich isotherm, i.e., the CR adsorption was a monolayer on heterogeneous sites of the CTAB-capped perovskite. The calculated *q_m_* value was 151.52 mg·g^−1^. In a previous study, Ali and Al-Oufi reported the *q_m_* value of pure La_0.9_Sr_0.1_FeO_3_ as 13.89 mg·g^−1^ [26], indicating that CTAB modification enhanced the sorption performance of La_0.9_Sr_0.1_FeO_3_ for CR dye by approximately 10 times. Despite the decreased particle size, surface area, and porosity of the perovskite upon surfactant capping, the CTAB-capped sample showed a superior adsorption ability with respect to the pure perovskite.

The calculated value of the Langmuir constant, *b*, was 0.0058 L·mg^−1^, and the Freundlich constants, *K_f_* and *n*, were 0.99 and 1.09, respectively. The separation factor, *R_L_*, (dimensionless) can be calculated from the following equation [35]:

(5) $$R_{L}=\frac{1}{1+bC_{o}}$$

The value of *R_L_* can indicate whether the adsorption was favored or not, where *R_L_* = 0 (irreversible), 1 > *R_L_* > 0 (favored adsorption), *R_L_* = 1 (linear), and *R_L_* > 1 (unfavorable). The calculated *R_L_* value was 0.63, which indicated the favorable adsorption of CR dye on the CTAB-capped La_0.9_Sr_0.1_FeO_3_ perovskite.

#### 2.2.4. The Adsorption Mechanism 

**Scheme 1:**
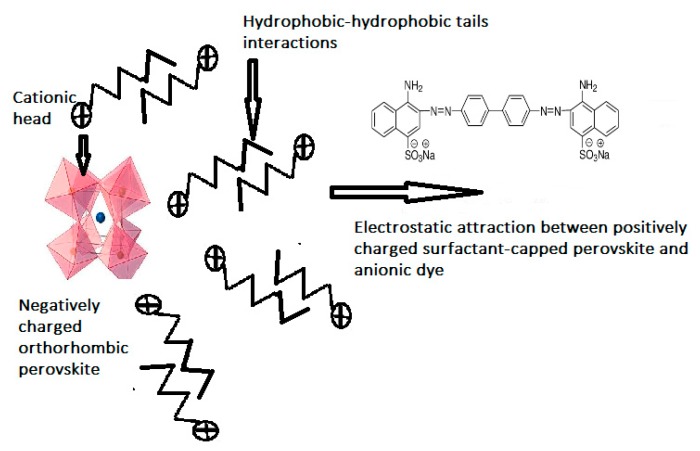
Represented CR Adsorption mechanism by CTAB-capped La_0.9_Sr_0.1_FeO_3_.

The proposed adsorption mechanism involved three steps: (1) the electrostatic attractions between the positively charged CTAB heads and the negatively charged perovskite surface, forming a monolayer of CTAB-capped perovskite with tails pointed outwards. (2) Formation of a surfactant bilayer through tail–tail hydrophobic interactions, with positively charged heads pointed outwards. (3) Strong electrostatic attractions between positively charged heads and negatively charged CR dye [37], Scheme 1.

In addition, a comparison with other CTAB-capped sorbents for CR dye is presented in Table 1. It can be shown that CTAB-capped La_0.9_Sr_0.1_FeO_3_ perovskite exhibited a considerable high-sorption performance. Although it did not show the highest *q_m_* value among the CTAB-modified materials presented, the proposed sorbent maintained its high performance at all pH values and showed a rapid dye uptake.

The sorption performance of the proposed sorbent for CR dye in the presence of a real sample matrix, taken from factory wastewater in Cairo, Egypt, was investigated. The calculated *q_m_* value was 143.23 mg·g^−1^; therefore, CTAB-capped perovskite maintained its excellent performance despite the matrix interferences. 

#### 2.2.5. Temperature Effect

**Figure 7:**
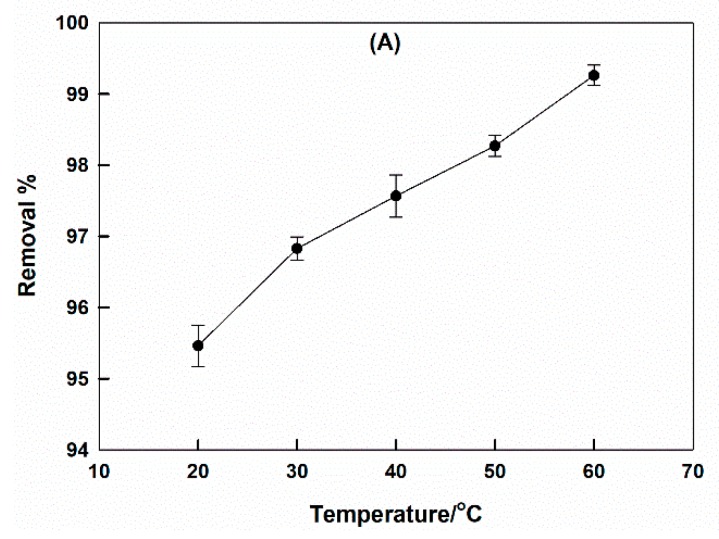

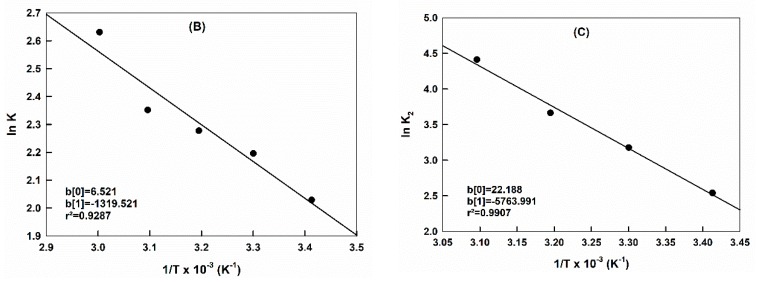
The relation between CR removal by CTAB-capped La_0.9_Sr_0.1_FeO_3_ with temperature; pH = 6, the dye concentration = 30 ppm, contact time = 1 h (**A**), transition-state (**B**), and Arrhenius (**C**) plots.

Figure 7A shows the dependence of the removal % of CTAB-capped La_0.9_Sr_0.1_FeO_3_ for CR on temperature. The removal % continued to increase with the temperature increase. 

Standard enthalpy and entropy changes, Δ*H^o^* and Δ*S^o^*, therefore, can be calculated by constructing the transition-state plot, according to the transition-state equation [35,45,46,47,48]:

(6) $$\ln K=\frac{{\Delta S}^{o}}{R}-\frac{{\Delta H}^{o}}{R}\left(\frac{1}{T}\right)$$

(7) $$K=\frac{C_{Ad}}{C_{e}}$$

where *K* was the equilibrium constant, *C_Ad_* and *C_e_* were concentrations of adsorbed dye on the sorbent and non-adsorbed dye in solution, respectively.

The transition-state plot is shown in Figure 7B. Calculated values of Δ*H^o^* and Δ*S^o^* were 10.95 kJ·mol^−1^ and 54.12 J·mol^−1^·K^−1^, respectively. This indicated that the adsorption is exothermic and the disorder is increased by the adsorption of CR on the CTAB-capped perovskite surface.

The value of the standard Gibbs free energy change, Δ*G^o^*, can be calculated using the following equation:

(8) $${\Delta G}^{o}={\Delta H}^{o}-{T\Delta S}^{o}$$

The calculated Δ*G^o^* value, was −4.91 kJ·mol^−1^, i.e., a spontaneous adsorption at room temperature.

The value of the activation energy, *E_a_*, can be calculated from the slope of the Arrhenius plot, which is shown in Figure 7C. The value was found to be 47.84 kJ·mol^−1^, which indicated that the adsorption of CR on CTAB-capped La_0.9_Sr_0.1_FeO_3_ perovskite is a chemisorption—it involved an electrostatic attraction between the positively charged sorbent and the negatively charged dye [26].

#### 2.2.6. The Sorption Performance by Repeated Use and its Regeneration

**Figure 8:**
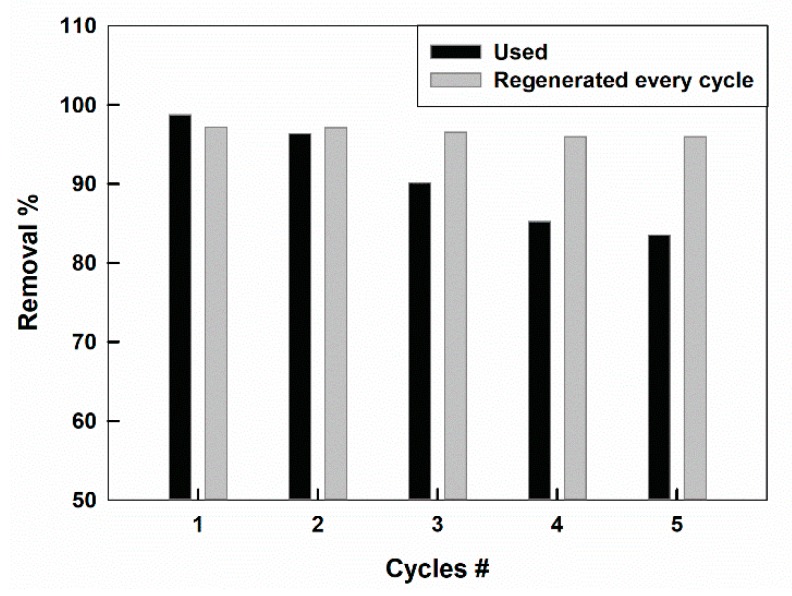
The removal % of (black) used and (gray) regenerated CTAB-capped La_0.9_Sr_0.1_FeO_3_ as a function of the number of use cycles.

CTAB-capped La_0.9_Sr_0.1_FeO_3_ perovskite was used for several cycles to examine its sorption ability with repeated use. Figure 8 shows the change of the removal % for CR dye with the number of use cycles, indicated by the black columns. The removal % is decreased from 98.8 to 83.5% after the 5th cycle, indicating a removal efficiency decrease by about 15.5% after five operating cycles. This reflected an acceptable performance of CTAB-capped La_0.9_Sr_0.1_FeO_3_ sorbent. 

A possible sorbent regeneration can be performed by stirring the used sorbent in 1 mmol L^−1^ of aqueous surfactant solution for 1 h, before each use. The change of the removal % with the number of use cycles for the regenerated sorbent, is shown by the gray columns in Figure 8. It can be shown that the regenerated sorbent maintained its excellent performance even after five cycles of use, as indicated by the unchanged removal % values.

## 3. Materials and Method

### 3.1. Materials

La(NO_3_)_3_·6H_2_O (99.9%), Fe(NO_3_)_3_·9H_2_O (99.9%), Sr(NO_3_)_2_ (99.9%), and C_6_H_8_O_7_ (99%), NH_4_OH (33%), HNO_3_ (65%), and CTAB (99%) were purchased from Sigma Aldrich. CR dye, C_32_H_22_N_6_Na_2_O_6_S_2_ (Brixworth, Northants, United Kingdom). All chemicals were used as-received. 

### 3.2. Microwave-assisted Citrate Combustion Synthesis of La_0.9_Sr_0.1_FeO_3_ Perovskite 

La(NO_3_)_3_·6H_2_O, Sr(NO_3_)_2_, and Fe(NO_3_)_3_·9H_2_O were mixed in a molar ratio of 0.9:0.1:1.0, and dissolved in distilled water. Citric acid was added to the metal ion solution, the pH value of which was previously adjusted to 8, at the same ratio to that of the total metal ions. The mixture was heated until combustion occurred in a microwave oven (700 watt for 30 min.). The resultant black powder was calcined at 900 °C for 3 h [26]. CTAB capping was performed by immersing the final calcined powder in 1 mmol L^−1^ of aqueous CTAB solution for 1 h with stirring.

### 3.3. Adsorption Test

A quantity of 0.05 g CTAB-capped perovskite/25 mL dye solution was shaken at 150 rpm for 1 d at an ambient temperature. The solution was then centrifuged at 3500 rpm for 1h. The concentration of the remaining, non-adsorbed dye was calculated by measuring the absorbance of clear solution by the UV-Vis spectrometer (Evolution 300, United Kingdom) at λ_Max_ of 466 nm.

The removal % of the CR dye can be estimated according to the following equation [44,45,46,47,48]:

(9) $$\mathrm{Removal}\;\%=\frac{C_{o}-C_{e}}{C_{o}}\times 100$$

The adsorbed CR amount, at the equilibrium, *q_e_* (mg g^−1^), was calculated from the following:

(10) $$q_{e}=\frac{\left(C_{o}-C_{e}\right)V}{W}$$

where *C_o_* and *C_e_* were the initial and equilibrium concentrations of the dye (mg L^−1^), V was the solution volume (L) and W was the sorbent mass (g).

Uncertainties of adsorption experiment parameters were listed in Table S1.

### 3.4. Characterization Instruments

X-ray diffractograms were used for the phase identification (XRD-7000, Shimadzu), at 40 kV and 30 mA, using a CuK_α_ incident beam (λ = 0.154 nm). FTIR spectroscopy was used for identification of characteristic functional groups, (IRAffinity-1S, Shimadzu).

Scanning electron microscopy was used to examine the surface morphology (Superscan SS-550, Shimadzu), with an accelerating voltage = 25 kV. 

Micromeritics ASAP 2020 was used to evaluate Brunauer–Emmet–Teller (BET) surface area values, with N_2_ adsorption isotherms at −196 °C at a relative pressure (P/P_o_) of 0.2. 

## 4. Conclusions

La_0.9_Sr_0.1_FeO_3_ can be successively capped with a cationic surfactant CTAB. The capping did not alter the perovskite chemical structure but resulted in a larger particle size with decreased porosity and BET surface area. CTAB-capped La_0.9_Sr_0.1_FeO_3_ can be used as an excellent sorbent for anionic CR dye, due to the formation of an oppositely charged dye–CTAB complex. The proposed sorbent has many advantages over reported CTAB-capped sorbents; it maintained its high performance at any pH value, showed fast dye uptake, and the removal % was high—approximately 97% at the start of the adsorption. The adsorption followed the Freundlich isotherm, which indicated a monolayer chemical adsorption of CR dye on heterogeneous CTAB-capped perovskite sites. The calculated *q_m_* value was 151.52 mg·g^−1^, which was 10 times higher than that of the pure perovskite. The calculated *E_a_* value was 47.84 kJ·mol^−1^, reflecting a chemical surface reaction. CTAB-capped La_0.9_Sr_0.1_FeO_3_ also showed an unaffected performance in the presence of the sample matrix, *q_m_* = 143.23 mg·g^−1^, which highly recommended it for applications in the field. It can be easily regenerated with unchanged removal ability, thus, offering an economic benefit. 

## Figures and Tables

**Table 1:** A comparison of the sorption performance of CTAB-capped La_0.9_Sr_0.1_FeO_3_ with reported CTAB-modified materials for the CR removal.

Sorbent	*q_m_* (mg·g^−1^)	Optimum pH Conditions	Equilibrium Time/min.	Reference
CTAB-chitosan beads	94.4	Acidic	240	[35]
CTAB-chitosan hydrogel beads	433.1	Acidic	240	[36]
CTAB-Tea waste	106.4	Independent	30	[37]
CTAB-Hectorite	182.0	Independent	120	[38]
CTAB-Kaolin	24.5	Alkaline	10	[39]
Bentonite-CTAB	210.0	Independent	90	[40]
graphene oxide-CTAB	2767.0	Acidic	60	[41]
**CTAB-La_0.9_Sr_0.1_FeO_3_**	**151.5**	**Independent**	**Less than 5**	**This work**

## Supplementary Materials

The following are available online, Table S1: Uncertainties of adsorption experiment parameters.
