# Supplementary material for: The Sorption Performance of Cetyl Trimethyl Ammonium Bromide-Capped La0.9Sr0.1FeO3 Perovskite for Organic Pollutants from Industrial Processes
Source: Molecules. 2020 Apr 2;25(7):1640. doi: 10.3390/molecules25071640 (PMC7180810; doi:10.3390/molecules25071640)
Supplement: Supplementary file 1 [file molecules-25-01640-s001.pdf]

Table S1: Uncertainties of adsorption experiment parameters.

Parameter	Error	Remark
Material (dye and sorbent) mass	± 0.1 mg	Balance uncertainty
Solution preparation and dilution	± 0.02 µL<br>± 0.05 mL	Pipette error<br>Measuring flask error
pH adjustment	± 0.02	Standard error of pH meter
Temperature	± 0.4 °C	Temperature controller error
Absorbance measurement	0.25 %	Standard uncertainty percent
Calibration curve fitting	1.28 %	Standard uncertainty percent
